# Shifted T Helper Cell Polarization in a Murine *Staphylococcus aureus* Mastitis Model

**DOI:** 10.1371/journal.pone.0134797

**Published:** 2015-07-31

**Authors:** Yanqing Zhao, Ming Zhou, Yang Gao, Heyuan Liu, Wenyu Yang, Jinhua Yue, Dekun Chen

**Affiliations:** College of Veterinary Medicine, Northwest A&F University, Yangling, Shaanxi Province, People’s Republic of China; University of Michigan Health System, UNITED STATES

## Abstract

Mastitis, one of the most costly diseases in dairy ruminants, is an inflammation of the mammary gland caused by pathogenic infection. The mechanisms of adaptive immunity against pathogens in mastitis have not been fully elucidated. To investigate T helper cell-mediated adaptive immune responses, we established a mastitis model by challenge with an inoculum of 4 × 10^6^ colony-forming units of *Staphylococcus aureus* in the mammary gland of lactating mice, followed by quantification of bacterial burden and histological analysis. The development of mastitis was accompanied by a significant increase in both Th17 and Th1 cells in the mammary gland. Moreover, the relative expression of genes encoding cytokines and transcription factors involved in the differentiation and function of these T helper cells, including *Il17*, *Rorc*, *Tgfb*, *Il1b*, *Il23*, *Ifng*, *Tbx21*, and *Il12*, was greatly elevated in the infected mammary gland. IL-17 is essential for neutrophil recruitment to infected mammary gland via CXC chemokines, whereas the excessive IL-17 production contributes to tissue damage in mastitis. In addition, a shift in T helper cell polarization toward Th2 and Treg cells was observed 5 days post-infection, and the mRNA expression of the anti-inflammatory cytokine *Il10* was markedly increased at day 7 post-infection. These results indicate that immune clearance of *Staphylococcus aureus* in mastitis is facilitated by the enrichment of Th17, Th1 and Th2 cells in the mammary gland mediated by pro-inflammatory cytokine production, which is tightly regulated by Treg cells and the anti-inflammatory cytokine IL-10.

## Introduction

Mastitis is one of the most prevalent diseases in dairy ruminants worldwide, with an incidence ranging from 10 to 74% in dairy cows and 9 to 50% in dairy goats [1–4]. Mastitis also occurs in other animals, including camel, buffalo, cats and dogs [5–8]. Mastitis causes considerable economic losses due to decreases in the quantity and quality of milk as well as early culling of dairy animals and high treatment costs, which are estimated to be $2 billion annually in the US alone [9]. To minimize this economic damage and maintain animal health, antibiotic treatments are important for mastitis therapy [10]. However, public health concerns, including antibiotic residues in food for human consumption and the occurrence of drug-resistant bacteria, have increased interest in the development of immunotherapy for mastitis. Active immunization with vaccines, a strategy that is dependent on the immune system to trigger protective immune responses, only ameliorates the clinical signs of mastitis and does not have a clinically meaningful ability to prevent new infections [11, 12]. In addition, cytokine immunotherapy based on recombinant bovine cytokines such as IL-2, IFN-γ and TNF-α do not induce any protective effect against coliform or *Staphylococcus aureus* (*S*. *aureus*) mastitis [13]. Such failures can be attributed primarily to the limited knowledge of the subtle changes in the cytokine milieu of the mammary gland that accompanies mastitis. Thus, a better understanding of mammary gland immunology is needed to explore effective immunotherapeutic approaches to mastitis.

Mastitis is an inflammation of the mammary gland. The udder features a specific breast anatomy with the production of a variety of immune cells and soluble factors for defense against invading pathogens [14, 15]. Milk macrophages, resident leukocytes and epithelial cells are the first cells to encounter and recognize bacterial pathogens entering the mammary gland through the teat canal. Neutrophils are then heavily recruited from the blood into the infected mammary gland, where they recognize, phagocytize, and kill the invading pathogens at the early stage of infection [16, 17]. Adaptive immunity plays an important role in immune clearance when innate defenses fail to completely eliminate mastitis-causing pathogens. B cell production of opsonizing antibodies that enhance neutrophil phagocytosis must be stimulated to establish a host adaptive immune response against pathogens. In addition to cytotoxic T lymphocytes, large numbers of T helper (Th) lymphocytes migrate into the infected udder and orchestrate effective adaptive immune responses [18–20]. These cell subsets can release chemokines and inflammatory cytokines, such as CXCL10, CCL2, CCL20, IL-17, IL-12, IFN-γ, IL-1β, IL-6, TGF-β and IL-10, which are significantly increased in the milk of dairy ruminants with mastitis [21, 22]. These cytokines are not only critical for the maintenance of a local inflammatory environment but also contribute to the differentiation of distinct T helper cells. However, the specific T helper cell subsets, including Th1, Th2, Th17 cells and regulatory T cells (Treg), that are mobilized in mastitis are not well defined, although it had been emphasized by Sordillo et al. that enhance mucosal cellular immunity and trafficking of memory T cells to the mammary gland are needed as a strategy to develop effective immunization against specific mastitis-causing pathogens [23].

To investigate T helper cell-mediated adaptive immunity in mastitis, we established a mouse model of *S*. *aureus* mastitis, which has been used previously to study the pathogenesis and control of bovine mastitis [24, 25]. T helper cell polarization and the cytokines involved in the differentiation and function of distinct T helper cell subsets were assessed in this murine model by flow cytometry and quantitative real-time PCR (qRT-PCR), respectively. The results obtained in the present study expand our current understanding of mammary gland immunity and lay the foundation for future immunotherapy of mastitis in dairy ruminants.

## Materials and Methods

### Mice

Wild-type C57BL/6 mice were purchased from the College of Medicine, Xi’an Jiaotong University (China). Lactating mice were used at 16–20 weeks of age. For IL-17 neutralization, 250 μg anti-mouse IL-17A antibody (clone TC11-18H10.1, Biolegend, San Diego, CA, USA) or isotype control was injected intraperitoneally 1 day prior to infection. Recombinant mouse IL-17A (PeproTech, Rocky Hill, NJ) was injected concurrently with bacterial infection, one dose of 1 μg per mammary gland, at the site of infection. All mice were housed in a specific pathogen-free facility and treated in accordance with the guidelines of the Care and Use of Laboratory Animals of the Ministry of Health, China. The study was approved by the Research Ethics Committee of Northwest A&F University.

### *S*. *aureus* infection

*S*. *aureus* isolated from milk samples of dairy goats with mastitis in China was used to establish a mouse model of experimental mastitis as described previously [26]. Prior to challenge, a single isolated colony from a Luria Bertani (LB) agar plate was inoculated into 4 ml of LB medium and incubated with shaking at 220 rpm at 37°C for 6 h. After centrifugation at 2,000 × *g* for 5 min, the bacterial pellet was washed, resuspended and diluted in sterile phosphate-buffered saline (PBS) at a density of 4 × 10^7^ colony-forming units (CFUs)/ml for intramammary infection.

The mouse model of mastitis was induced as described previously [24, 26]. Briefly, 7- to 10- day-old pups were removed from lactating mice to allow milk accumulation in the mammary gland for 1–2 h before bacterial inoculation. Next, the lactating mice were anesthetized via i.p. injection of ketamine (100 mg/kg body weight, Diamondback Drugs, Arizona, USA), and each gland of the fourth pair of mammary glands as counted from head to tail (on the left and right, L4 and R4) was inoculated separately with 0.1 ml of the prepared inoculum containing 4 × 10^6^ CFUs or 1 × 10^6^ CFUs of *S*. *aureus* using a 32-gauge blunt needle. Control mice were inoculated with PBS. After inoculation for 6 h, the mammary glands of the lactating mice were suckled by the returned offspring.

### Quantification of *S*. *aureus* burden in the mammary gland

At the indicated time points after *S*. *aureus* infection, the lactating mice were euthanized, and whole L4 mammary glands were harvested and homogenized in 1 ml of PBS using a tissue grinder under sterile conditions. The homogenates and their serial log dilutions were quickly plated onto LB agar plates and incubated for 24 h at 37°C. The number of CFUs remaining in each gland was then evaluated.

### Histological analysis

To assess tissue destruction and inflammatory cell infiltration at different times post-infection, excised R4 mammary glands were fixed in 4% paraformaldehyde, embedded in paraffin, serially sectioned and stained with hematoxylin and eosin. Tissue slices were examined by light microscopy (original magnification × 400).

### Flow cytometry

Peripheral blood mononuclear cells (PBMCs) were isolated using standard protocols in lymphocyte separation medium (Huajing, Shanghai, China). In brief, spleens were harvested, ground, and passed through a sterile 200-μm-pore metal sieve. The resulting cell suspension was layered over an equal volume of Ficoll density gradient solution and centrifuged. The recovered cells were washed and resuspended at a density of 1 × 10^6^ cells/ml in RPMI 1640 medium (HyClone, Beijing, China) supplemented with 10% fetal bovine serum (FBS, Gibco, Australia), 100 U/ml penicillin, 100 μg/ml streptomycin, 2 mM glutamine, 10 mM HEPES, 1 mM sodium pyruvate, and 50 μM 2-ME. A trypan blue dye exclusion assay was used to evaluate cell viability. Lymphocytes were isolated from the mammary gland as previously described with some modifications [26, 27]. The tissues were harvested, washed and cut into small pieces. The prepared samples were incubated in Ca^+^- and Mg^+^-free Hanks’ balanced salt solution containing 1 mM dithiothreitol and 5 mM EDTA with slow shaking for 30 min, followed by passage through a sterile 200-μm-pore metal sieve. After centrifugation, the cell pellet was resuspended in RPMI 1640 medium, and lymphocytes were isolated as previously described in Ficoll density gradient solution.

To analyze intracellular cytokines, the isolated lymphocytes were stimulated with PMA (50 ng/ml, Sigma-Aldrich, St. Louis, MO, USA) and ionomycin (1 μg/ml, Sigma-Aldrich) in the presence of brefeldin A (10 μg/ml, Sigma-Aldrich) for 6 h. Surface staining was performed using FITC anti-CD4 antibody (Ab), APC anti-CD25 Ab, and FITC anti-CD8 Ab. The samples were then fixed and permeabilized, and intracellular cytokines were stained using the following mouse antibodies: PE anti-IL-17A, APC anti-IFN-γ, and Percp/cy5.5 anti-IL-4. Finally, the labeled cells were washed and resuspended in 0.2 ml of cell staining buffer. Isotype controls were included in this experiment. Antibodies and buffers were purchased from Biolegend unless otherwise indicated, and all procedures were performed according to the manufacturer’s instructions. Data were acquired using a FACSAria flow cytometer (BD Biosciences, San Jose, CA, USA) and analyzed using FlowJo software (Tree Star, Ashland, OR, USA).

### Quantitative real-time PCR

Total RNA was extracted from the mammary gland using RNAiso Plus and chloroform. After quantification using a NanoDrop ND-2000 spectrophotometer (Thermo Fisher Scientific, Wilmington, DE, USA), up to 1 μg of total RNA was reverse-transcribed to cDNA using the PrimeScript RT reagent kit with gDNA eraser. Quantitative real-time PCR was performed using SYBR Premix Ex Taq II on an iQ5 Real-Time PCR Detection System (Bio-Rad, Hercules, CA, USA). The primers used in this experiment are listed in S1 Table; GAPDH was used as a housekeeping gene. All reagents were purchased from TaKaRa (Dalian, China), and all procedures were performed according to the manufacturer’s protocol. The data are expressed as fold change using the 2^-ΔΔCt^ method.

### Statistical analysis

GraphPad Prism software (v. 6. 0, GraphPad Software Inc., San Diego, CA, USA) was used for statistical analyses and graphing. Differences between experimental and control groups were assessed using *t*-tests or one-way ANOVA with Dunnett test. P < 0.05 was considered significant.

## Results

### Mouse model of *S*. *aureus* mastitis

**Fig 1:**
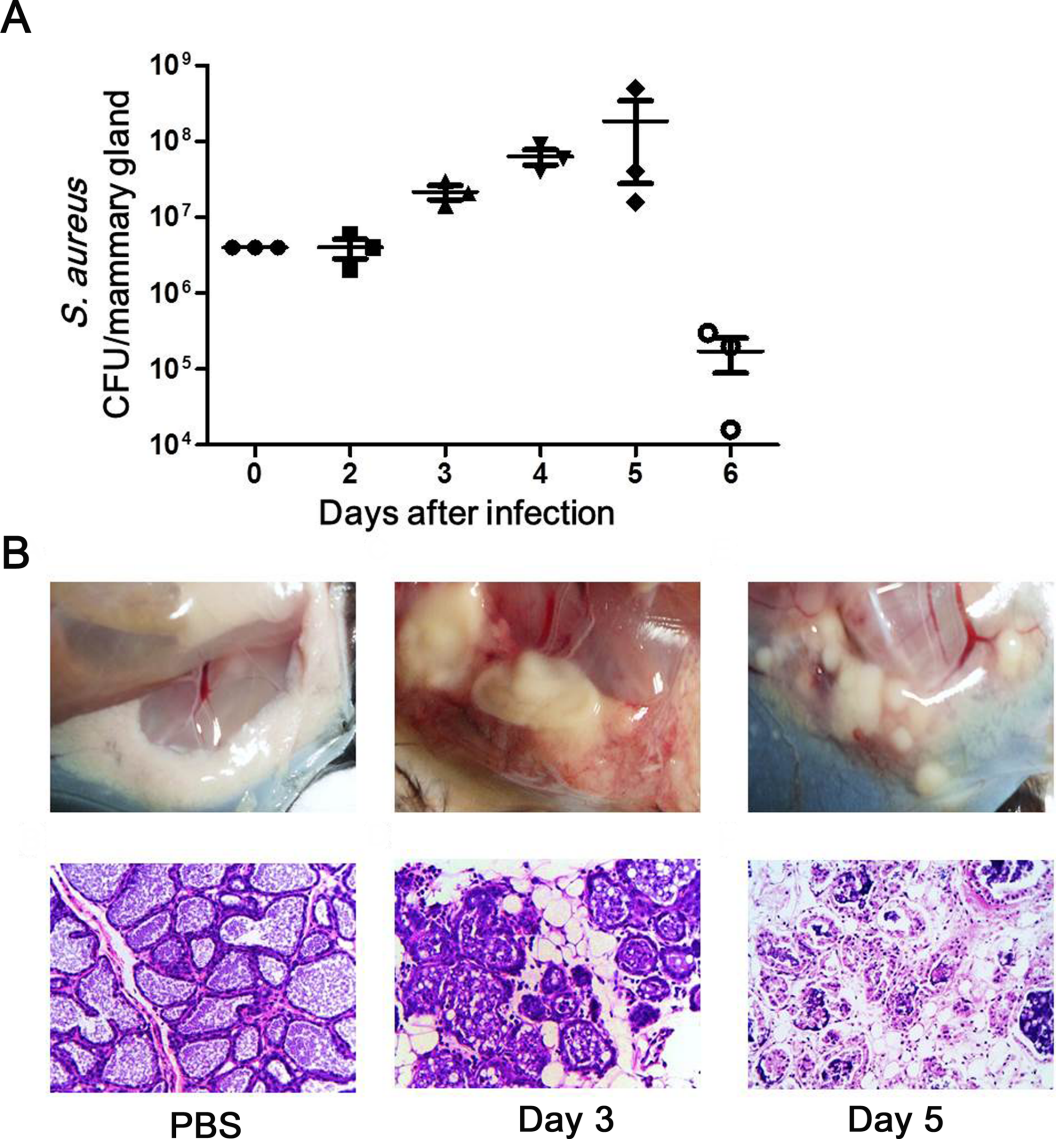
Assessment of murine mastitis model. Lactating mice were inoculated with PBS or 4 × 10^6^ CFUs of *S*. *aureus* in the mammary gland and euthanized at the indicated times after infection. (A) Dynamics of S. aureus burden in a murine mastitis model. The mammary glands were harvested, homogenized, and quantitatively cultured *in vitro*. Each dot represents one mouse. (B) Histopathological changes in the mammary gland in a murine mastitis model. The mammary glands were harvested, fixed and stained with hematoxylin and eosin (original magnification × 400). The data shown are representative of three independent experiments.

To investigate the adaptive immune responses against mastitis-causing pathogens, a mastitis model was established in lactating mice. After challenge with a *S*. *aureus* inoculum, the bacterial burden in the mammary glands of infected mice increased, peaking at day 5 post-inoculation, and decreasing significantly thereafter (Fig 1A). An approximately 45-fold increase in the bacterial number was observed in the mammary gland at day 5 post-challenge compared with the initial injection quantity, and *S*. *aureus* was not completely cleared from the mammary gland after 6 days of infection. In addition, tissue degeneration and pustules were observed in the infected mammary gland (Fig 1B). Histologically, alveolar atrophy and epithelial and luminal cell discontinuities were observed in the tissue sections, accompanied by monocyte and neutrophil infiltration in the alveolar lumen post-infection (Fig 1B).

### Induction of Th17/Th1 cells and pro-inflammatory cytokines in murine *S*. *aureus* mastitis

**Fig 2:**
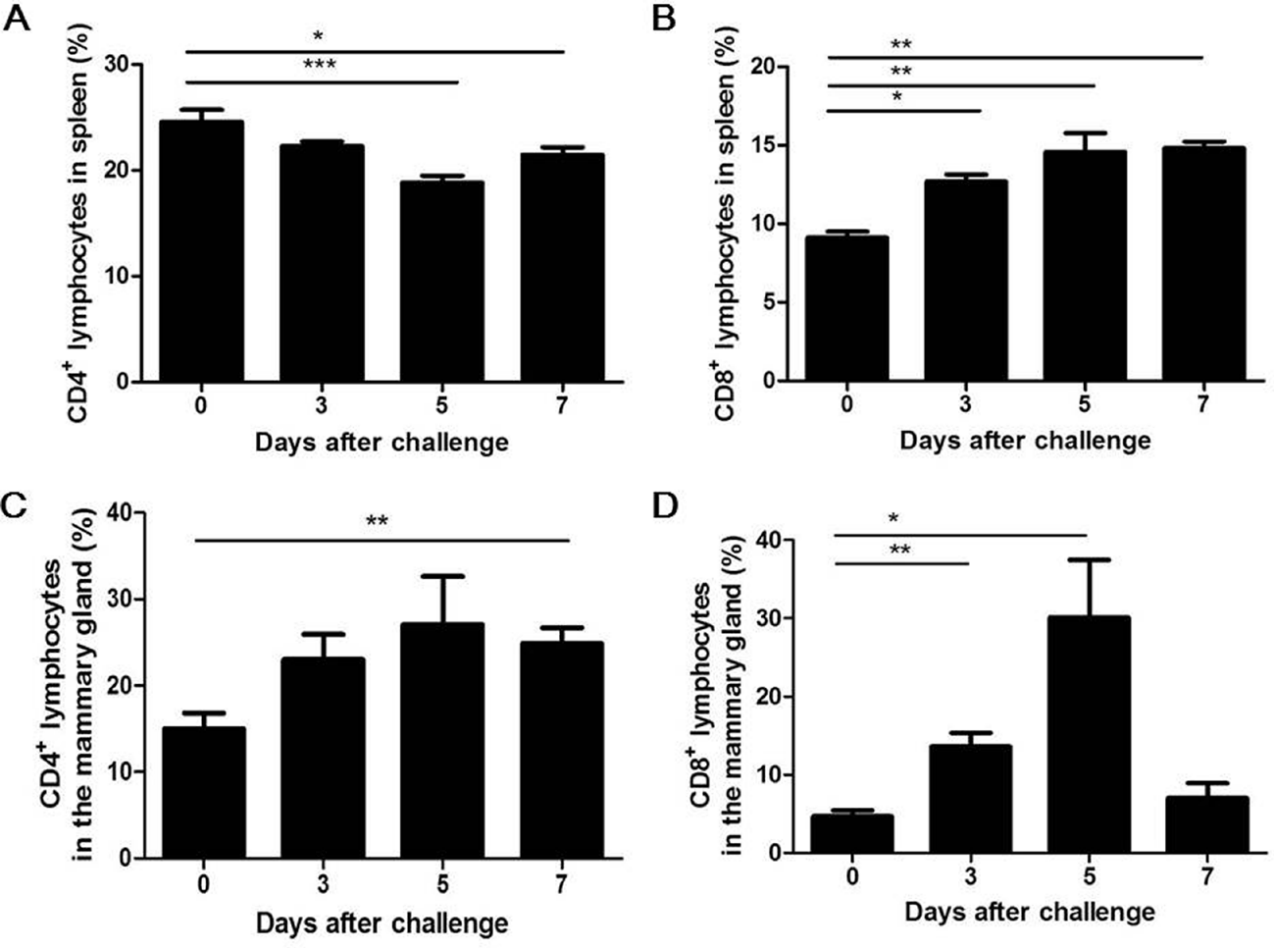
Both T helper and T killer cells are induced in response to *S*. *aureus* intramammary infection. Lactating mice were challenged with 4 × 10^6^ CFUs of *S*. *aureus* in the mammary gland. At the indicated times, PBMCs were prepared, and cell surface markers were stained for flow cytometry analysis. The percentages of CD4^+^ lymphocytes in the spleen (A) and mammary gland (C) are indicated. The percentages of CD8^+^ lymphocytes in the spleen (B) and mammary gland (D) are indicated. The data shown are the mean ± SEM (n = 6) of three representative experiments with similar results. * p < 0.05, ** p < 0.01, *** p < 0.001.

In the mastitis model, the presence of T helper and T killer cells confirmed the involvement of T cells in the adaptive immune response against *S*. *aureus* infection in the udder. The percentages of T helper cells differed between the spleen and mammary gland in the infected mice. In the spleen, T helper cells were significantly decreased at 5 days post-challenge (Fig 2A); however, in the mammary gland, they increased markedly at day 7 post-challenge (Fig 2C). The percentages of T killer cells were significantly higher in both the spleen and mammary gland at 3 days post-infection (Fig 2B and 2D), and the highest percentage of T killer cells (30.00%) was detected in local inflammatory lesions at day 5 post-challenge. Taken together, these data indicate that both T helper and T killer cells contribute to the adaptive immune response against *S*. *aureus* infection in a murine model of mastitis.

**Fig 3:**
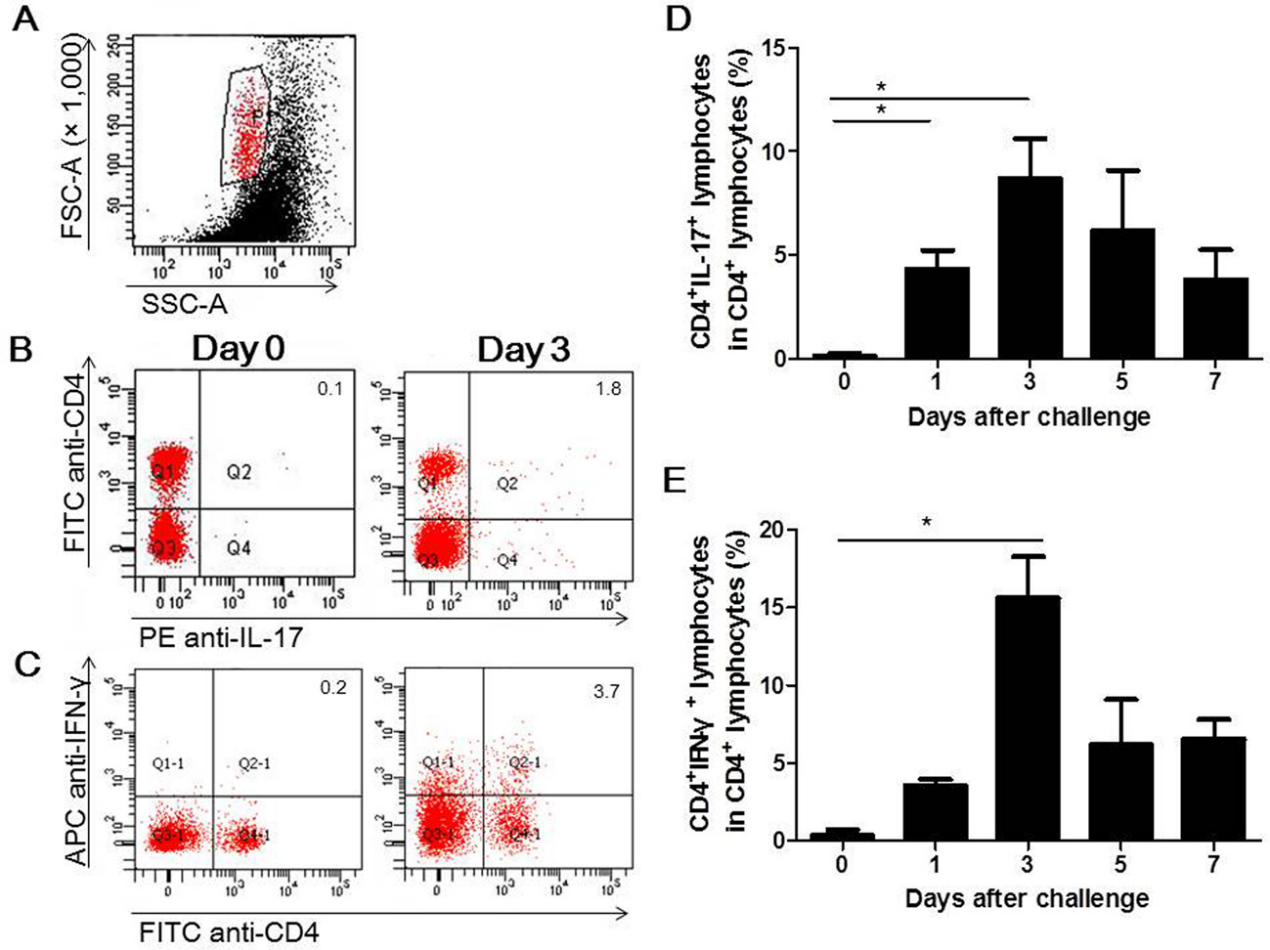
Recruitment of Th17 and Th1 cells in the mammary gland after *S*. *aureus* intramammary infection. PBMCs from the mammary glands of mice infected with 4 × 10^6^ CFUs of *S*. *aureus* were collected, stained and analyzed by flow cytometry. Representative flow cytometry results for Th17 cells (B) and Th1 cells (C). The percentages of positive cells are shown in each panel. The histograms present the percentages of Th17 cells (D) or Th1 cells (E) among the CD4^+^ cells. The values are the mean ± SEM (n = 6) of three representative experiments with similar results. * p < 0.05.

**Fig 4:**
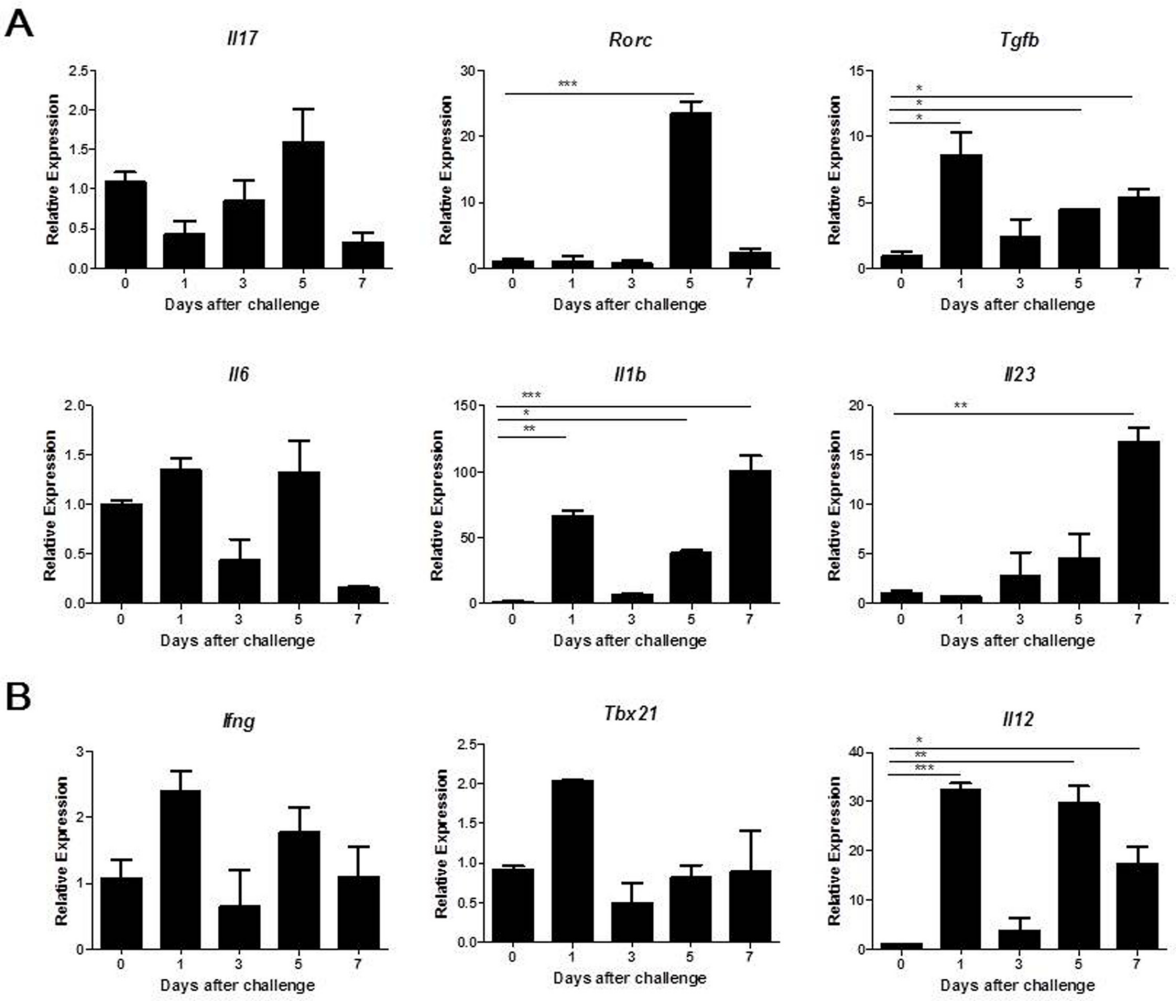
Characterization of pro-inflammatory cytokine production and transcription factors expression in the mammary gland following *S*. *aureus* infection. Lactating mice were challenged with 4 × 10^6^ CFUs of *S*. *aureus* in the mammary gland, and mammary tissue was collected for analysis by qRT-PCR at the indicated time points. (A) Relative expression of cytokines and transcription factors associated with the differentiation and function of Th17 cells. (B) Relative expression of genes encoding cytokines and transcription factors involved in Th1 differentiation and function. The data are expressed as the mean ± SEM of one experiment representative of three experiments with similar results with six mice per group. * p < 0.05, ** p < 0.01, *** p < 0.001.

Because T helper cells were present in the mammary glands of mice infected with *S*. *aureus*, the percentages of distinct CD4^+^ T helper cell subsets were analyzed by flow cytometry. Th17 (CD4^+^IL-17^+^) cells were detected at day 1, peaked at day 3 post infection and then decreased gradually. The increase in Th17 cells was significant, with this cell type accounting for as many as 8.69% of total CD4^+^ lymphocytes (Fig 3D). Similar dynamics were observed in the change in Th1 (CD4^+^IFN-γ^+^) cells, which also peaked at day 3 after challenge and accounted for 15.63% of the total CD4^+^ lymphocytes (Fig 3E). Because Th17 and Th1 cells were elevated in infected mice, we investigated whether these cell subset-related cytokines were generated in the mammary glands of infected mice. As shown in Fig 4A, the relative expression levels of *Tgfb*, *Il1b* and *Il23*, which previous studies have demonstrated contribute to the differentiation of Th17 cells [28, 29], were significantly higher at different times after *S*. *aureus* infection compared to the PBS control. The mRNA level of *Il6*, another cytokine that participates in Th17 cell differentiation, was not markedly changed. Although the expression of the Th17 cell master transcription factor *Rorc* was significantly increased at day 5 post-infection, the mRNA level of *Il17* was only slightly increased post-infection. In addition, the expression levels of *Ifng* and its key transcription factor *Tbx21* were only slightly modulated. IL-12, which promotes Th1 cell differentiation, was significantly induced in response to *S*. *aureus* infection (Fig 4B).

### Th2 cells associated with immunity to *S*. *aureus* in the mammary gland

**Fig 5:**
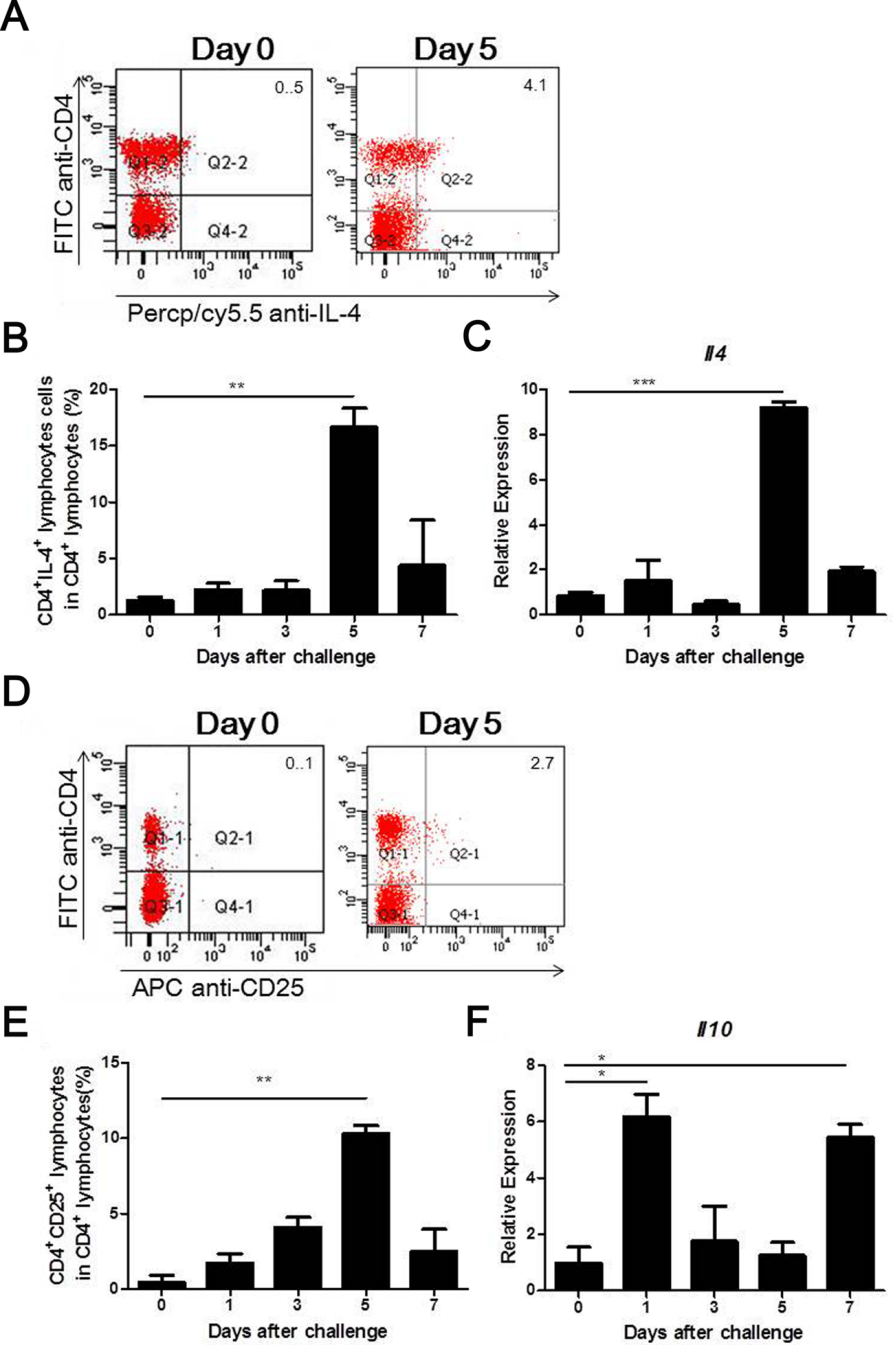
Recruitment of IL-4-producing Th2 cells and Treg cells to the infected mammary gland. Lactating mice were administered an intramammary injection of 4 × 10^6^ CFUs of *S*. *aureus*. Representative FACS results for Th2 cells (A) and Treg cells (D). The percentage of positive cells is shown in each panel. The proportion of IL-4-producing Th2 cells (B) or Treg cells (E) among the CD4^+^ cells was analyzed by flow cytometry. The expression of IL-4 (C) and IL-10 (F) in the infected mammary gland was analyzed by qRT-PCR. Experiments were performed in triplicate with six mice in each group. The data are expressed as the mean ± SEM. * p < 0.05, ** p < 0.01, *** p < 0.001.

As shown in Fig 5B, the proportion of Th2 (CD4^+^IL-4^+^) cells remained low at 3 days post-infection but then significantly increased and reached a maximum (16.66% of total CD4^+^ lymphocytes) at 5 days post-challenge. Consistent with the presence of Th2 cells, the mRNA expression of its prototypical cytokine *Il4* increased significantly at 5 days post-infection (Fig 5C).

### Treg cells regulate immune responses against *S*. *aureus* infection in the mammary gland

We investigated whether Treg cells are recruited to the mammary gland to prevent excessive inflammatory responses against *S*. *aureus* infection. As shown in Fig 5E, the proportions of Treg (CD4^+^CD25^+^) cells gradually increased and peaked at 10.32% of total CD4^+^ lymphocytes at 5 days post-infection. In addition, the mRNA level of *Il10*, a potent anti-inflammatory cytokine, increased significantly at days 1 and 7 after challenge, respectively (Fig 5F).

### Role of IL-17 in the mammary gland immunity against *S*. *aureus* infection

**Fig 6:**
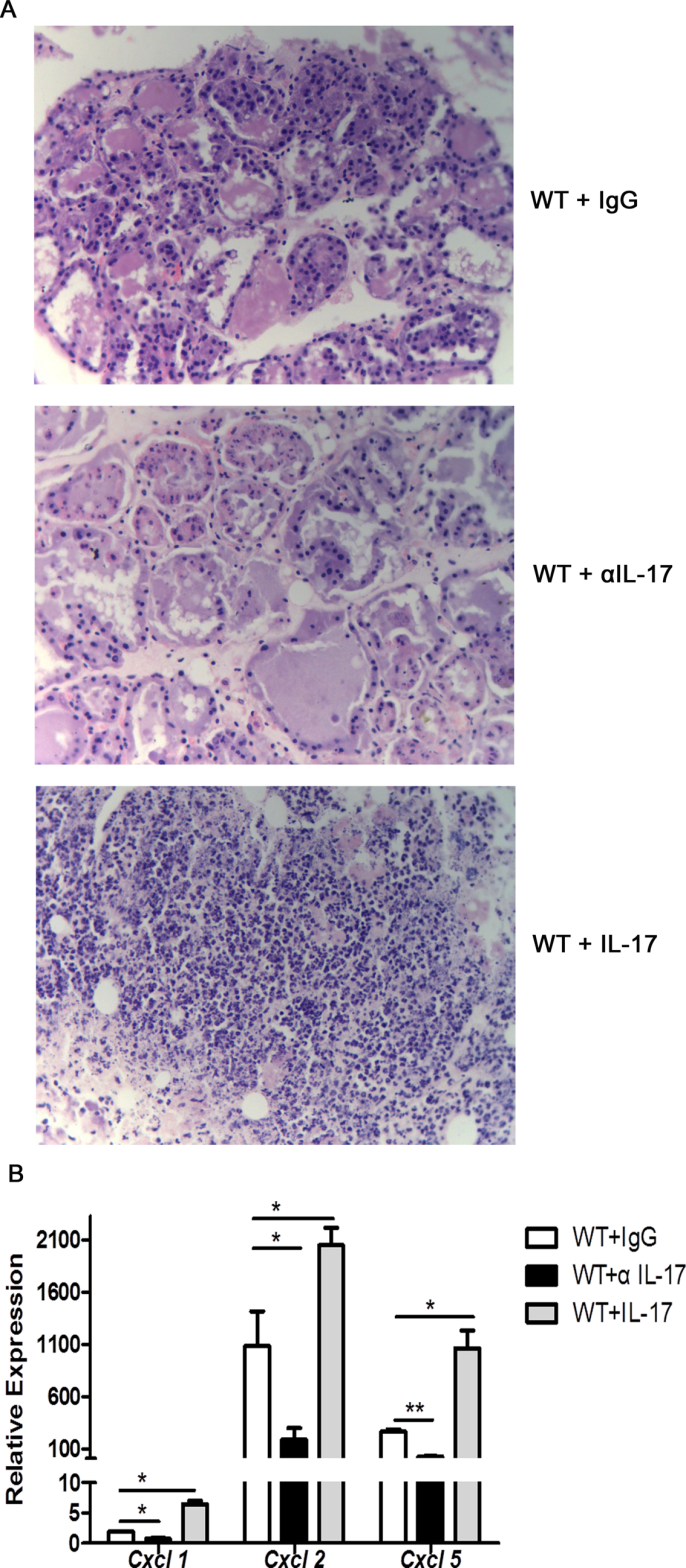
Il-17 enhances neutrophil infiltration through expression of CXC chemokines. Lactating mice were administered an intramammary injection of 1 × 10^6^ CFUs of *S*. *aureus*, and the mammary gland of the mice were harvested after 30 hours of infection. Anti-mouse IL-17A antibody (250 μg per mouse) or isotype control was injected intraperitoneally 1 day prior to infection. Recombinant mouse IL-17A was injected concurrently with bacterial infection, one dose of 1 μg per mammary gland, at the site of infection. (A) Histological analysis was performed to detect the inflammatory cell infiltration in the infected mammary gland by H&E staining. Representative photomicrography of sections is shown at a 400 times the original magnification. (B) The relative expression of CXCL1, CXCL2 and CXCL5 in the infected mammary gland was analyzed by qRT-PCR. These results are representative of three independent experiments (n = 3) and are presented as mean ± SEM. * p < 0.05, ** p < 0.01.

To further explore the role of IL-17 in host defense against *S*. *aureus* intramammary infection, we focused on neutrophil-recruiting chemokines that are the key target genes of IL-17, such as CXCL1, CXCL2 and CXCL5. As shown in Fig 6, treatment with anti-IL-17 antibody decreased inflammatory cell infiltration in the infected mammary gland, and significantly decreased the mRNA expression of *Cxcl1*, *Cxcl2* and *Cxcl5* when compared with IgG control. Conversely, administration of recombinant IL-17 to WT mice at the time of bacterial infection resulted in a significant increase in mRNA expression of *Cxcl1*, *Cxcl2* and *Cxcl5*. As well as, the lactating mice receiving the IL-17 treatment exhibited severe structural damage to alveolar and a dramatically increased inflammatory cell infiltration, particularly neutrophils.

## Discussion

*S*. *aureus* mastitis is found commonly in dairy ruminants with subclinical or chronic infections. Antibiotic resistance, which may be explained by biofilm formation and existence of *S*. *aureus* within epithelial cells and in macrophages, is associated with this pathogenic disease. Immune-based therapies seem to be an alternative to antibiotic treatment for control of *S*. *aureus* induced mastitis. *S*. *aureus* vaccines that induced primarily antibody responses demonstrated poor protection for infection in the mammary gland, whereas T cell mediated cellular responses that play an important role to enhance PMN function and need to be considered for a successful vaccine in mucosal vaccination against mastitis [30]. The cytokines in the prevention and/or treatment of *S*. *aureus* mastitis will exhibit promising results in future, if extensive studies focused on the changes in the cytokine profile in *S*. *aureus* mastitis have been performed [13]. In this study, adaptive immunity, particularly the CD4^+^ cell-mediated adaptive immune responses, was investigated at the cellular and molecular levels in the murine *S*. *aureus* mastitis model. Here we show that both Th1 and Th17 cells were activated during infection progression, as the dynamics of Th2 and Treg cells were monitored in the recovery stage of mastitis. Moreover, the relative expression of genes encoding cytokines and transcription factors involved in the differentiation and function of various T helper cells was greatly elevated in the infected mammary gland.

As a critical pro-inflammatory cytokine, IL-17 not only plays an important role in the pathogenesis of multiple autoimmune diseases but also mediates the immune responses against infection by various extracellular microbes [28, 31]. Upregulation of *Il17* mRNA has been detected in tissues from the alveolar, ductal, gland cistern and teat canal regions of the bovine mammary gland at 48 h post-infection with *S*. *aureus* [32]. IL-17 is also highly expressed in the milk somatic cells of dairy cows with *S*. *aureus* mastitis [33]. At the protein level, IL-17 peaks at 24 or 48 h in the milk of dairy goats following challenge with *E*. *coli* or *S*. *aureus*, respectively [34]. In the present study, *Il17* mRNA increased on day 5 post-intramammary infection with *S*. *aureus*, although it did not change significantly in persistent mastitis. We previously detected γδ T cells, an important source of IL-17, at the early stage of *S*. *aureus* mastitis in mice [26]. In the present study, we demonstrated for the first time that Th17 cells participate in *S*. *aureus*-induced mastitis. A significant increase in the percentage of Th17 cells as well as the inflammatory milieu for Th17 differentiation were observed. Cytokines involved in Th17 differentiation, including TGF-β, IL-1β and IL-23, were greatly increased in the mammary gland following *S*. *aureus* intramammary infection. This finding is consistent with the results of previous studies demonstrating that these cytokines are markedly increased in the mammary glands and milk of diary ruminants infected with mastitis-causing pathogens [35]. How IL-17 orchestrates an effective inflammatory response in the mammary gland is unclear. In this study, IL-17 administration exacerbates neutrophil infiltration through the induction of CXCL1, CXCL2 and CXCL5 following *S*. *aureus* intramammary infection, whereas neutralization of the IL-17 by anti-IL-17 antibody reduced neutrophil infiltration. Consistently, Bougarn et al suggested that recombinant bovine IL-17 stimulates *in vitro* the IL-17 receptor complex, including IL-17RA and IL-17RC, expressed in bovine mammary epithelial cells to produce a variety of antibacterial proteins, cytokines, and chemokines, which target neutrophils and mononuclear leukocytes [36]. Based on this previous work and our results, we propose that after *S*. *aureus* intramammary infection, IL-17 produced by γδ T cells and Th17 cells activates mammary epithelial cells to release various soluble factors, particularly chemokines, which recruit neutrophils and mononuclear leukocytes to the infected mammary gland to enhance host clearance of pathogens. IL-17 immunity to enhance PMN infiltration into mammary gland has been suggested as a potential strategy for design of a mucosal vaccine against *S*. *aureus*, which needs further investigation.

Th17 cells expand the classical Th1/Th2 paradigm. Th1 cells play an important role in host defense against intracellular parasitic infections, promoting the activation and proliferation of cytotoxic lymphocytes, natural killer cells and macrophages via the production of IFN-γ, TNF-β and IL-2. Consistent with previous studies, in the present study, a slight but detectable increase in IFN-γ secreted by Th1 cells was observed following *S*. *aureus* intramammary infection, resulting in increased opsonization and phagocytosis by neutrophils via class switching to the IgG2 isotope [14, 35]. Although *S*. *aureus* is now recognized as a facultative intracellular pathogen, it is also extensively distributed in the extracellular space [37]. Coincidentally, we observed a significant increase in Th2 cells, which drive antibody-mediated immunity against *S*. *aureus* intramammary infection. Prenafeta et al observed that antibody titers against a slime-associated antigenic complex were significantly enhanced in sera and milk of dairy cows after vaccination with *S*. *aureus* biofilm-embedded bacteria, and the concentration of *S*. *aureus* in milk was greatly reduced compared with the control groups following challenge with a heterologous strain of *S*. *aureus* [38]. Thus, both cellular and humoral immunity are involved in the immune clearance of *S*. *aureus* from the udder.

However, in contrast to the recruitment of Th17/Th1 cells at 3 days post-infection, Th2 cells and the prototypical cytokine IL-4 were significantly increased at 5 days after inoculation in the present study. A two-day delay in the recruitment of Th2 cells to the mammary gland was observed. A shift in the T-cell population from a Th17/Th1 phenotype toward Th2 subsets was observed during mastitis. This skewing of the immune system toward Th2 cells appears to be protective against the exacerbated inflammatory response accompanied by *S*. *aureus* clearance, consistent with the results of previous studies [39]. Chen et al suggested that Th2-biased immune responses were essential for limiting acute tissue damage during experimental helminth infection [40]. Within 2 days after invasion by *Nippostrongylus brasiliensis* larvae, IL-17 was rapidly elevated in lung tissue, and IL-17-dependent neutrophil recruitment contributed to acute lung damage. By 4 days after inoculation, however, IL-4 and IL-13 increased significantly, resulting in the inhibition of IL-17 expression and control of inflammation via the overexpression of *Il10*. Furthermore, IL-10 has been shown to strictly restrain IL-17 production to generate efficient protective immunity without inducing a pathological response [41, 42]. Slight et al demonstrated that increased Th1 and Th17 responses were induced in IL-10-deficient mice after infection with a live vaccine strain of *Francisella tularensisi*; however, the mortality of the mice was also increased due to exacerbated IL-17 production, increased neutrophil recruitment and associated lung pathology [43]. Consistently, *Il10* mRNA expression was significantly increased on day 7 after *S*. *aureus* intramammary challenge in the present study. Furthermore, Treg cells, one of the primary sources of IL-10, were markedly accumulated in the mammary gland at 5 days after inoculation. In summary, Th1, Th2 and Th17 cells are all activated for efficient immunity against *S*. *aureus* infection in a murine mastitis model but are tightly regulated by Treg cells and IL-10.

Notably, on day 1 after *S*. *aureus* infection, most cytokines, including IL-6, IL-1β, IFN-γ, IL-12, TGF-β and IL-10, were enhanced to varying degrees. This phenomenon suggests that not only adaptive but also innate immune responses are evoked within hours of infection and play a critical role in the clearance of pathogens during mastitis.

In conclusion, the present findings demonstrate that exposure to *S*. *aureus* in the mammary gland of lactating mice induces the activation of multiple T helper subsets and that the balance between immunopathology and protective immunity is tightly regulated. To develop effective prevention and treatment of mastitis in a clinical setting, these findings should be further validated in dairy ruminants because of the significant differences between species. The results of the current study based on a murine model enhance our understanding of immune defense mechanisms against mastitis-causing pathogens.

## Supporting Information

S1 Table Primers used for real-time PCR in this study. (DOCX)
