# Supplementary material for: Shifted T Helper Cell Polarization in a Murine Staphylococcus aureus Mastitis Model
Source: PLoS One. 2015 Jul 31;10(7):e0134797. doi: 10.1371/journal.pone.0134797 (PMC4521801; doi:10.1371/journal.pone.0134797)
Supplement: S1 Table — (DOCX) [file pone.0134797.s001.docx]

**Supporting Information**

**S1 Table. Primers used for real-time PCR in this study.**

| **Gene** | **GenBank accession no.** | **Sequence (5’-3’)** | **Amplicon size (bp)** | **Reference** |
| --- | --- | --- | --- | --- |
| *Gapdh* | NM_008084 | F: ACCTGCCAAGTATGATGAC | 119 | [1] |
|  |  | R: GGGAGTTGCTGTTGAAGT |  |  |
| *Il17a* | NM_010552 | F: CTCCAGAATGTGAAGGTC | 89 | [1] |
|  |  | R: GAACGGTTGAGGTAGTCT |  |  |
| *Il1b* | NM_008361 | F: GAATCTATACCTGTCCTGTGTAA | 130 |  |
|  |  | R: GCTCTTGACTTCTATCTTGTTG |  |  |
| *Il12a* | NM_001159424 | F: CTGCTTGACTCTGACATCT | 108 |  |
|  |  | R: CCACTGCTGACTAGAACTC |  |  |
| *Il6* | NM_031168 | F: AGA AGGAGTGGCTAAGGA | 187 |  |
|  |  | R: GAGAACAACATAAGTCAGATAC |  |  |
| *Tbx21* | NM_019507 | F: ACCAGAGCGGCAAGTGGG | 69 | [2] |
|  |  | R: TGGACATATAAGCGGTTCCCTG |  |  |
| *Tgfb1* | NM_011577 | F: TGACGTCACTGGAGTTGTACGG | 169 | [3] |
|  |  | R: GGTTCATGTCATGGATGGTGC |  |  |
| *Il10* | NM_010548 | F: AAGGACCAGCTGGACAACAT | 172 | [4] |
|  |  | R: TCTCACCCAGGGAATTCAAA |  |  |
| *Ccr6* | NM_009835 | F: GGTTCATCTCCATCATCATCT | 81 |  |
|  |  | R: GCTCACAGACATCACGAT |  |  |
| *Il4* | NM_021283 | F: TCAACCCCCAGCTAGTTGTC | 199 | [5] |
|  |  | R: CGAGCTCACTCTCTGTGGTG |  |  |
| *Rorc* | XM_005357024 | F: CCGCTGAGAGGGCTTCAC | 241 | [6] |
|  |  | R: TGCAGGAGTAGGCCACATTACA |  |  |
| *Il21* | NM_021782 | F: ATCCTGAACTTCTATCAGCTCCAC | 196 | [7] |
|  |  | R: GCATTTAGCTATGTGCTTCTGTTTC |  |  |
| *Ifng* | NM_008337 | F: ATCTGGAGGAACTGGCAAAA | 246 | [5] |
|  |  | R: TGAGCTCATTGAATGCTTGG |  |  |
| *Il23a* | NM_031252 | F: AGCGGGACATATGAATCTACTAAGAGA | 244 | [8] |
|  |  | R: GTCCTAGTAGGGAGGTGTGAAGTTG |  |  |
| *Cxcl1* | NM_008176 | F: GCTGGGATTCACCTCAAGAA | 180 | [9] |
|  |  | R: TCTCCGTTACTTGGGGACAC |  |  |
| *Cxcl2* | NM_009140 | F: CCAAGGGTTGACTTCAAGAAC | 92 | [10] |
|  |  | R: CCCTTGAGAGTGGCTATGACT |  |  |
| *Cxcl5* | NM_009141 | F: AGCTGCCCCTTCCTCAGTC | 100 | [11] |

**Supplemental References**

1. Jing XQ, Cao DY, Liu H, Wu XY, Zhao XD, Chen DK. Pivotal role of IL-17-producing γδ T cells in mouse chronic mastitis experimentally induced with *Staphylococcus aureus*. Asian J Anim Vet Adv. 2012;7: 1266-1278.

2. Harrington LE, Hatton RD, Mangan PR, Turner H, Murphy TL, Murphy KM, et al. Interleukin 17-producing CD4+ effector T cells develop via a lineage distinct from the T helper type 1 and 2 lineages. Nat Immunol. 2005;6: 1123-1132.

3. Casteels K, Waer M, Bouillon R, Depovere J, Valckx D, Laureys J, et al. 1,25-Dihydroxyvitamin D3 restores sensitivity to cyclophosphamide-induced apoptosis in non-obese diabetic (NOD) mice and protects against diabetes. Clin Exp Immunol. 1998;112: 181-187.

4. Zhang X, Edwards JP, Mosser DM. Dynamic and transient remodeling of the macrophage IL-10 promoter during transcription. J Immunol. 2006;177: 1282-1288.

5. Schröppel B, Zhang N, Chen P, Zang W, Chen D, Hudkins KL, et al. Differential expression of chemokines and chemokine receptors in murine islet allografts: the role of CCR2 and CCR5 signaling pathways. J Am Soc Nephrol. 2004;15: 1853-1861.

6. Ivanov II, McKenzie BS, Zhou L, Tadokoro CE, Lepelley A, Lafaille JJ, et al. The orphan nuclear receptor RORgammat directs the differentiation program of proinflammatory IL-17+ T helper cells. Cell. 2006;126: 1121-1133.

7. Zhou L, Ivanov II, Spolski R, Min R, Shenderov K, Egawa T, et al. IL-6 programs T(H)-17 cell differentiation by promoting sequential engagement of the IL-21 and IL-23 pathways. Nat Immunol. 2007;8: 967-974.

8. Uhlig HH, McKenzie BS, Hue S, Thompson C, Joyce-Shaikh B, Stepankova R, et al. Differential activity of IL-12 and IL-23 in mucosal and systemic innate immune pathology. Immunity. 2006;25: 309-318.

9. Glass WG, Subbarao K, Murphy B, Murphy PM. Mechanisms of host defense following severe acute respiratory syndrome-coronavirus (SARS-CoV) pulmonary infection of mice. J Immunol. 2004;173: 4030-4039.

10. Brown HJ, Lock HR, Sacks SH, Robson MG. TLR2 stimulation of intrinsic renal cells in the induction of immune-mediated glomerulonephritis. J Immunol. 2006;177: 1925-1931.

11. Andreasen C, Carbonetti NH. Pertussis toxin inhibits early chemokine production to delay neutrophil recruitment in response to *Bordetella pertussis* respiratory tract infection in mice. Infect Immun. 2008;76: 5139-5148. doi: 10.1128/IAI.00895-08.
